# Development and Validation of a Sitting-Balance Confidence Scale for Individuals with Spinal Cord Injury

**DOI:** 10.3390/healthcare14172841

**Published:** 2026-09-03

**Authors:** J. P. Barfield, Jacqueline M. LaBarbera, Kelly Werts, Kaida Lou, Yinghao Pan

**Affiliations:** 1 College of Health and Human Services, University of North Carolina at Charlotte, Charlotte, NC 28223, USA; 2 Carolinas Rehabilitation Hospital, Charlotte, NC 28203, USA; jackie.labarbera@advocatehealth.org (J.M.L.); kelly.werts@advocatehealth.org (K.W.); 3 Department of Mathematics and Statistics, University of North Carolina at Charlotte, Charlotte, NC 28223, USA; klou2@charlotte.edu (K.L.); ypan8@charlotte.edu (Y.P.)

**Keywords:** disability, functional independence, paraplegia, tetraplegia

## Abstract

**Highlights:**

**What are the main findings?**

- A novel interview-based Patient-Reported Sitting Balance Confidence (PR-SBC) scale was developed for therapists to evaluate non-ambulatory individuals with spinal cord injury, incorporating both daily and participation-based activities.
- The PR-SBC demonstrated promising inter-rater reliability, internal consistency, and validity evidence relative to a related falls concern measure.

**What are the implications of the main findings?**

- The PR-SBC captures psychological aspects of balance (confidence and self-efficacy) not addressed by existing performance-based or fear-focused tools.
- This interview and evaluation tool can enhance clinical assessment and guide targeted interventions to improve participation in community, sport, and physical activity.

**Abstract:**

**Background/Objectives:** Participation in sport and daily activities among individuals with spinal cord injury (SCI) is influenced not only by physical capacity but also by psychological factors such as balance confidence. Existing measures primarily assess performance or fear of falling, leaving a gap in evaluating how perceived balance impacts activity choice. This study aimed to (1) develop a Patient-Reported Sitting Balance Confidence (PR-SBC) interview instrument to enable therapists to assess and evaluate non-ambulatory individuals with SCI, (2) incorporate participation-based activities relevant to perceived confidence in community and sport activities, and (3) evaluate its psychometric properties. **Methods:** Instrument development used a Delphi process with clinicians, researchers, individuals with SCI, and care partners to create and refine items. The final 19-item PR-SBC was administered to 31 individuals with SCI (inpatient and outpatient). Inter-rater reliability was assessed using intraclass correlation coefficients (ICC), internal consistency via Cronbach’s alpha, and validity through correlation with the SCI Falls Concern Scale (SCI-FCS). Exploratory regression analyses examined demographic and clinical predictors of PR-SBC scores. **Results:** The PR-SBC demonstrated promising inter-rater reliability (*ICC* = 0.96) and internal consistency (α = 0.931). A moderate inverse correlation with SCI-FCS scores was also observed (*r* = −0.51). Outpatient setting and manual wheelchair use were associated with higher balance confidence, while injury-related variables were not significant predictors. **Conclusions:** The PR-SBC is a potential interview instrument for assessing sitting balance confidence in individuals with SCI. By capturing perceived balance across functional and participation contexts, it complements existing measures and may support improved clinical decision-making, targeted interventions, and enhanced participation outcomes.

## 1. Introduction

The International Classification of Functioning, Disability, and Health (ICF) framework conceptualizes participation as the interaction between health conditions, body functions and structures, and contextual factors that influence engagement in life activities across home, work, and community environments [1,2]. Sport and physical activity represent critical domains of participation, as they are strongly associated with quality of life, employment, social integration, and interpersonal interaction among individuals with disabilities, including those with spinal cord injury (SCI) [3,4,5,6,7,8,9,10]. Following SCI, impairments in body functions and structures result in significant challenges to participation, with environmental and personal factors mediating the degree to which individuals engage in meaningful activities.

Among these factors, balance and fall-related concerns have emerged as critical determinants of participation and quality of life. Balance plays a foundational role in functional independence among individuals with SCI, particularly for those who are non-ambulatory. Sitting balance is essential for self-care tasks, transfers, and wheelchair propulsion, as well as for participation in sport and physical activity. Impairments in postural control, trunk stability, and sensorimotor integration following SCI compromise seated balance, affecting the performance of activities of daily living (ADLs), transfers, wheelchair mobility, and overall independence. Objective measures of sitting balance are well-established in SCI rehabilitation [11,12]. A range of performance-based assessments—such as the Sitting Balance Scale, Function in Sitting Test (FIST), modified Functional Reach Test, SitBAT, and Sitting Balance Measure—quantify sitting balance capacity during ADLs. Evidence demonstrates moderate-to-strong associations between sitting balance, wheelchair skills, and functional independence, as measured by the Spinal Cord Independence Measure (SCIM III) [13,14]. Additionally, wheelchair skills confidence and mobility-related independence are linked to improved quality of life outcomes, particularly in physical and environmental domains [15].

Psychological constructs—including balance confidence, perceived stability, and concern about falling—have received comparatively limited attention until recently [16,17,18,19]. Traditional balance measures emphasize observed performance and independence rather than perceived balance ability. For example, the SCIM III, while widely validated, evaluates task completion and assistance levels but does not capture confidence, perceived stability, or fear associated with balance-related activities. As a result, psychological factors that may influence participation—particularly in higher-level or discretionary activities such as sport and physical activity—remain underrepresented. This gap is clinically relevant, as discrepancies between actual ability and perceived confidence may increase fall risk or contribute to activity restriction. Specifically, individuals with low confidence may unnecessarily restrict activity despite adequate ability, whereas those with high confidence but insufficient capacity may be at increased risk for falls.

To address psychological aspects of sitting balance, the Spinal Cord Injury Falls Concern Scale (SCI-FCS) was developed to assess fear of falling during basic daily activities [20]. The SCI-FCS demonstrates strong psychometric properties and remains the primary SCI-specific measure of fall-related concerns. However, several limitations restrict its broader applicability. Notably, consistent floor effects suggest limited sensitivity for higher-functioning or community-active individuals [21,22,23,24]. Furthermore, the scale focuses primarily on low-risk ADL activities and does not adequately capture higher-level balance challenges associated with sport, recreation, or community participation [21]. Importantly, concern about falling represents only one dimension of balance-related perception and does not fully encompass constructs such as balance confidence, perceived control, and self-efficacy. Prior research indicates that fear of falling and task-specific balance confidence are related but distinct constructs, underscoring the need for more comprehensive assessment approaches.

Examined collectively, existing measurement tools do not fully capture the multidimensional nature of sitting balance in SCI. Performance-based assessments quantify physical capacity but omit perceived ability, while current self-report tools focus narrowly on fear of falling and lack sensitivity across functional levels. Sitting balance confidence, defined as an individual’s belief in their ability to maintain stability and safely engage in activities, represents a distinct and clinically meaningful construct that may influence participation independently of physical performance.

Accordingly, there is a need for a comprehensive, SCI-specific measure that captures sitting balance confidence across a spectrum of functional and participation-based activities. Such a tool would complement existing assessments by providing insight into the psychological factors that influence engagement in daily life and physical activity. Therefore, the purpose of this study was to: (1) develop a Patient-Reported Sitting Balance Confidence (PR-SBC) interview instrument to enable therapists to assess and evaluate non-ambulatory individuals with SCI, (2) incorporate participation-based activities relevant to perceived confidence in community and sport activities, and (3) evaluate its psychometric properties in both inpatient and outpatient populations.

## 2. Materials and Methods

### 2.1. Participants

Instrument Creation. We recruited 19 participants as part of a Delphi Method to create an interview instrument. We initially contacted researchers and inpatient clinicians at rehabilitation hospitals, persons living with SCI, and care partners to persons with SCI. This work was initiated from a CARF-accredited spinal cord injury site and experts were delimited to clinicians and researchers at this level of expertise. Of the 19 participants, 8 submitted feedback by requested timelines. The final expert panel, following two rounds of review, consisted of 3 clinicians (including one professional with a dual researcher/clinical role), 3 individuals living with SCI, and 2 care partners of persons living with SCI. Delphi participants reported 18 ± 11 years of experience in their current profession and 20 ± 10 years of experience working with individuals with physical impairments.

Instrument Evaluation. We assessed 16 inpatient and 17 outpatient clients with the finalized instrument whose demographics are included in Table 1. Inclusion criteria included individuals 18 years of age or older who were living with a neurological impairment, were not ambulatory, and had no current therapy goals for ambulation. Exclusion criteria included inadequate cognition or communication ability as determined by the research team. Thirty-two out of 33 participants had a spinal cord injury; therefore, we delimited the sample to only individuals with SCI. Additionally, one participant was removed because of highly discrepant scores among two therapists (sample outlier), resulting in a total sample of *N* = 31.

### 2.2. Instrument

The research team conducted a literature review on established sitting balance confidence measures and created a draft interview instrument with 16 outcome questions. The tool was designed to enable therapists to assess and evaluate balance confidence across multiple settings (inpatient, outpatient, community). The instrument was structured to align with the Activity Balance Confidence Scale which is one of the core outcome measures recommended by the Academy of Neurologic Physical Therapy; however, it only addresses ambulatory individuals. After 2 rounds of Delphi Method review, the final Patient Reported-Sitting Balance Confidence Scale (PR-SBC; Appendix A) contained 19 items anchored from 1 (not at all confident) to 10 (completely confident).

Scoring was designed so that therapists can assess balance confidence across multiple life participation tasks, document tasks that are/are not relevant for a patient based on their function, and identify the amount of support or assist needed for each task. The PR-SBC reflects average confidence across total items scored, allowing clinicians to evaluate the same metric regardless of how many tasks are/are not relevant to rehabilitation and post-rehabilitation goals (community engagement). Scoring also enables comparison to future normative data (e.g., comparison to AIS functional classification; setting type). The final instrument includes ADLs as well as life participation activities (e.g., leisure activities, work activities, social interaction) organized into home/clinic and community-based settings. Unique to falls confidence assessments, the PR-SBC allows therapists to drive the administration, enabling clinicians to elicit complete responses and document assistance as needed to examine confidence under assisted and independent conditions.

### 2.3. Procedures

Instrument Creation. The original interview instrument was drafted internally by the research group (16 questions total). Following internal review by the CARF-accredited site clinic team, the initial draft was updated to include respondent demographic information (e.g., work experience in persons with physical impairments), home/clinic questions (*n* = 13 questions; exercise, complete household tasks), and community-based assessment options (*n* = 7; perform required work duties). The research team then distributed the Round 1 20-question interview instrument to Delphi participants with forced-choice response options for each item as either “important to include,” “less important to include,” or “should not include.” During this round of review, Delphi participants also had the option to recommend other potential test items and administrative recommendations (e.g., increase/decrease number of items, domains). Following Round 1, a total of 16 recommendations were received and integrated into a revised instrument, resulting in 19 outcomes addressing either home/clinic (*n* = 13) or community-based (*n* = 6) settings. This updated version was again sent to Delphi participants (Round 2) to review each item and suggest recommended administrative changes. Full agreement (i.e., all items ranked as “important to include;” no additional comments or suggested changes) was reached following Round 2 with the final version reported in Appendix A.

Instrument Evaluation. Inter-rater reliability was assessed by a primary and secondary therapist at the same SCI CARF accredited site. The lead therapist in the inpatient department has 36 years of experience and is a Board Certified Neurologic Clinical Specialist, and the lead outpatient therapist has 14 years of experience. The primary inpatient or outpatient therapist administered the PR-SBC to a research participant independently. Later in the same rehabilitation visit, a second administration was conducted by a secondary therapist. This method allowed therapists to conduct the interview and score participants separately to determine the extent that balance confidence could be measured consistently by different therapists (e.g., same visit, distinct administrations). Validity was assessed against the SCI Falls Concern Scale (SCI-FCS). Because these instruments measure distinct but related constructs, the current design is not a true criterion nor discriminant comparison. As such, the validity evidence for this comparison is exploratory in nature and important to instrument evaluation and development. For this comparison, the primary therapist administered the SCI-FCS during the same visit period as the PR-SBC.

### 2.4. Analysis

Reliability. ICC values were computed to examine inter-rater reliability for the PR-SBC for the sample as a whole and separately for inpatient and outpatient participants. Two-way absolute-agreement single-measure ICCs, reported as ICC(2,1), were used. Internal consistency was examined using Cronbach’s alpha across the 19 PR-SBC items using scores from Rater 1, the primary rater. Item diagnostics examined corrected item-total correlations, and alpha if item deleted.

Validity. Validity was examined using Pearson correlations between PR-SBC and SCI-FCS scores. The primary inpatient and outpatient therapist administered the SCI-FCS for the respective settings. Additionally, an exploratory multiple linear regression analysis examined demographic and clinical factors that may influence PR-SBC scores. The dependent variable was the PR-SBC score from Rater 1, and the independent variables included setting, time since injury, level of injury, SCI severity, and mobility mode. The independent variables were coded as follows: setting was coded as inpatient versus outpatient; time since injury was converted to approximate years; level of injury was coded as tetraplegia versus paraplegia; SCI severity was coded as AIS A/B versus AIS C/D; and mobility mode was coded as power wheelchair versus manual wheelchair. Because of the small sample size, results should be interpreted as exploratory. Given the exploratory and hypothesis-generating nature of the analyses, *p*-values were not adjusted for multiple testing.

## 3. Results

### 3.1. Descriptive Statistics and Regression Model

The PR-SBC score was calculated as the average of completed item responses rather than as a raw summed score. This approach was used because the instruments allow therapists to mark items as N/A if not a personal or therapeutic goal. By dividing the sum of completed item scores by the number of completed items, each participant’s final score remained on the original 1–10 response scale, allowing for more consistent comparisons across individuals with different numbers of completed items. Among the 31 participants included in the analysis, 28 had complete responses for all 19 Rater 1 items. The remaining three participants reported N/A for 1 item each and therefore had responses for 18 of the 19 items (94.7% completion). Thus, there were no participants with fewer than 18 completed items.

Descriptive statistics are reported in Table 2. In this preliminary sample, the mean PR-SBC Rater 1 item-mean score was 6.22 (SD = 1.90), with scores ranging from 2.53 to 9.05 (median = 6.16). Years since injury appeared right-skewed: most participants were relatively early post-injury, as reflected by a median of 0.83 years, while one participant was 50 years post-injury, which increased the mean to 3.45 years. In an exploratory bivariate analysis, years since injury was not significantly associated with PR-SBC Rater 1 item-mean score (*r* = 0.039, *p* = 0.834).

Additionally, the overall regression model was statistically significant, *F*(5, 25) = 2.889, *p* = 0.034, and explained 36.6% of the variance in PR-SBC Rater 1 score (*R*^2^ = 0.366; adjusted *R*^2^ = 0.240). After adjusting for the other predictors, outpatient setting and manual wheelchair use were associated with higher PR-SBC scores. Outpatient participants scored 1.51 points higher than inpatient participants (*p* = 0.027) and manual wheelchair users scored 1.53 points higher than power wheelchair users (*p* = 0.047). Time since injury, injury level, and SCI severity were not statistically significant predictors in the adjusted model (Table 3). All variance inflation factor (VIF) values were below 5, suggesting no evidence of problematic multicollinearity among the predictors.

The participant who reported 50-years post-injury was admitted to rehabilitation due to a second injury. To ensure that this individual was not an outlier to the regression model, we computed influence diagnostics and conducted a sensitivity analysis by refitting the regression model after excluding this case. This observation had high leverage (0.935) and a Cook’s distance of 3.672, indicating substantial influence on the fitted model. Additionally, the externally studentized residual was −1.25, suggesting that the participant’s PR-SBC score was not itself an extreme outcome value. The largest approximate DFBETA was −4.22 for years since injury, indicating that this observation strongly influenced the estimated coefficient for that predictor. However, the sensitivity analysis showed that some findings were not robust to the exclusion of this participant. The outpatient-setting estimate decreased from 1.51 (95% CI: 0.18 to 2.83; *p* = 0.027) to 1.00 (95% CI: −0.56 to 2.55; *p* = 0.199) and was no longer statistically significant (see Appendix B for comparison). The estimate for years since injury changed from approximately zero (*p* = 0.973) to 0.14 (*p* = 0.245), although it remained nonsignificant. In contrast, the manual-chair estimate remained relatively stable, changing from 1.53 (95% CI: 0.02 to 3.04; *p* = 0.047) to 1.58 (95% CI: 0.08 to 3.08; *p* = 0.039).

### 3.2. Reliability Evidence

Inter-rater reliability was promising. For the sample as a whole, ICC (2,1) was 0.96. Reliability was also sound among inpatient and outpatient participants (Table 4). Internal consistency of the PR-SBC was examined using Rater 1 scores. Internal consistency was clinically relevant for all comparison groups. Cronbach’s alpha was 0.931 for the sample, 0.843 for inpatient participants, and 0.951 for outpatient participants.

Corrected item-total correlations for Rater 1 scores were positive for all 19 items, ranging from 0.309 to 0.801. Alpha-if-deleted values ranged from 0.924 to 0.933, indicating that removing individual items *would not* meaningfully improve internal consistency. We identified “Travel in a vehicle” as having the lowest corrected item–total correlation (*r* = 0.309). Although this correlation is comparatively lower than those of the other items, it meets the commonly recommended minimum threshold of 0.30, indicating an acceptable, albeit modest, association with the remaining items. The relatively lower correlation may suggest that this item captures a somewhat distinct aspect of the underlying construct while still contributing meaningfully to the overall scale.

### 3.3. Validity Evidence

Validity against the SCI-FCS varied by setting (Table 5). Overall, the PR-SBC average score was moderately and significantly correlated with SCI-FCS. The relationship between the two scales was weaker among inpatient participants but still meaningful as a potential complementary interview instrument. The correlation between the two scales was strong among outpatient participants. As expected, the negative correlation coefficients reflect the expected scoring direction: higher PR-SBC scores indicate greater balance confidence, whereas higher SCI-FCS scores indicate greater concern.

## 4. Discussion

Falls are common among individuals with spinal cord injury (SCI), particularly among wheelchair users during transfers and mobility tasks, and are associated with injury, increased healthcare utilization, and reduced participation [25]. Importantly, perceived balance confidence and fear of falling influence behavior independently of physical ability. Individuals may restrict activity, avoid community participation, or decline rehabilitation challenges due to low confidence, even when objective balance performance is adequate [14,15,20,21,22,23,24]. These findings are consistent with the ICF framework, in which personal factors—such as perceived confidence—interact with body functions and environmental demands to influence participation in life activities.

This study addressed an important gap in rehabilitation settings by developing the Patient Reported-Sitting Balance Confidence (PR-SBC) scale to assess perceived balance confidence in individuals with SCI. The findings provide preliminary evidence supporting the reliability and validity of this interview instrument. Inter-rater reliability was promising, particularly in outpatient settings, suggesting that the PR-SBC can be administered consistently across raters. Additionally, moderate associations between the PR-SBC and the SCI Falls Concern Scale (SCI-FCS) reinforce that these instruments assess related but distinct constructs. Specifically, the SCI-FCS captures concern about falling, whereas the PR-SBC reflects broader balance confidence, including perceived stability and self-efficacy in performing functional and participation-based activities.

Exploratory analyses further suggest that contextual factors, including rehabilitation setting and mobility mode, may influence balance confidence. Outpatient participants and manual wheelchair users demonstrated higher balance confidence, potentially reflecting greater community exposure and functional independence. In contrast, time since injury, level of injury, and injury severity were not associated with PR-SBC scores after adjustment. However, these findings should be interpreted cautiously due to the relatively small sample size and exploratory nature of the analysis.

### 4.1. Integration with Existing Measures and Conceptual Models

Existing outcome measures in SCI rehabilitation capture important but incomplete aspects of sitting balance. Performance-based tools quantify physical capacity, and independence measures such as the SCIM III evaluate task completion and assistance levels [13,14]. However, these assessments do not capture perceived sitting balance ability, which may independently influence participation. Conversely, the SCI-FCS [20] represents an important advance in assessing psychological aspects of balance but is limited by its narrow focus on fear of falling and its reduced sensitivity among higher-functioning individuals.

Several SCI-FCS delimitations have been reported, including pronounced floor effects and relatively large “minimally detectable change” values ([23]; Table 6), which limit its responsiveness to change following rehabilitation. The literature documents scores clustered at the lower end of the scale across populations [21,22,23,24], suggesting minimal fall concerns among most participants, particularly those who are more active or adapted to wheelchair mobility. As a result, the SCI-FCS may function most effectively as a screening tool for individuals with high fall-related concern but may underestimate barriers among higher-functioning individuals. The PR-SBC may overcome this floor effect as sample scores ranged from 2.53 to 9.05 (median = 6.16), indicating the ability to capture a wide range of perceived confidence.

The PR-SBC was developed to address these existing scale limitations and better align with contemporary models of disability, including the ICF framework. By incorporating a wider range of activities that reflect real-world participation—such as work, travel, leisure, and social engagement—the PR-SBC extends beyond basic ADLs to capture balance confidence in meaningful life contexts. In addition, the use of a 10-point response scale enhances sensitivity to change, allowing the detection of subtle yet clinically meaningful differences over time and its measurement properties are promising for an initial instrument. The initial inter-rater reliability (*ICC* = 0.96), internal consistency reliability (0.93), and moderate validity evidence (*r* = −0.51) are all consistent with SCI-FCS psychometric properties (Table 6).

Findings from this study indicate that separate therapists can score balance confidence consistently from this tool and that all items contribute to the overall assessment. The PR-SBC can be used as a complementary sitting balance confidence scale. Observed differences between inpatient and outpatient participants in the current study are preliminary and more examination of this instrument is warranted. Importantly, the PR-SBC integrates multiple domains of functioning by linking sitting balance confidence to activity demands, environmental challenges, and participation goals. This approach reflects a more comprehensive conceptualization of balance within SCI, recognizing that participation is influenced not only by physical capacity but also by perceived ability and contextual barriers.

### 4.2. Clinical and Research Implications

The PR-SBC has several potential clinical applications. First, it provides a structured means of identifying low-confidence activities that may limit participation, enabling clinicians to target interventions more effectively. For example, reduced confidence in community-based tasks may highlight the need for skills training, adaptive equipment, or environmental modification. Second, the instrument facilitates patient-centered discussions regarding personal and environmental factors that influence sitting balance confidence, consistent with the ICF model. These may include physical barriers, social support, lifestyle factors, and prior experiences.

Additionally, the inclusion of information related to equipment use and assistance level enhances the interpretability of scores and supports the monitoring of change over time. Changes in confidence may reflect transitions in independence (e.g., reduced assistance or altered equipment), providing valuable insight into rehabilitation progress. The PR-SBC may also complement existing measures, such as the SCI-FCS and performance-based assessments, to provide a more comprehensive evaluation of fall risk and participation potential.

From a research perspective, the PR-SBC offers a tool to examine the role of sitting balance confidence as an independent determinant of participation, physical activity, and quality of life. Future studies should explore its responsiveness to intervention and its predictive validity in relation to falls, activity engagement, and long-term outcomes. Additional research can also examine test–retest reliability under conditions where the construct of balance confidence is stable (i.e., post-rehabilitation community participants).

### 4.3. Limitations

Several limitations should be considered. The sample size was modest relative to the number of variables examined, limiting statistical power and generalizability. As a result, analyses examining associations between PR-SBC scores and participant characteristics should be interpreted as exploratory. ICC values for inter-rater reliability must also be interpreted with caution as there is likely carryover effects from patient memory from two assessments in the same visit and selected regression coefficients—particularly the outpatient-setting and years-since-injury estimates—were sensitive to the influential observation. That being said, this study presents an initial starting point as estimates show promise of consistency across independent raters. Additionally, the cross-sectional design limits conclusions regarding causality or responsiveness to change. Further validation in more diverse samples—including individuals across different stages of recovery and functional levels—is warranted.

## 5. Conclusions

Balance confidence represents a critical yet underassessed determinant of participation among individuals with SCI. Consistent with the ICF framework, perceived confidence interacts with physical capacity and environmental factors to influence engagement in daily and community activities. The PR-SBC provides a novel, participation-oriented measure of sitting balance confidence with evidence of reliability and validity. By complementing existing performance- and fear-based assessments, this tool has the potential to enhance clinical evaluation, guide targeted interventions, and support improved participation and quality of life in individuals living with SCI.

## Figures and Tables

**Table 1:** Participant demographics (*N* = 31).

Variable		Sample	Inpatient	Outpatient
*n*		31	15	16
Age (yrs)		43 + 15	45 + 17	40 + 13
Time Since Initial Injury (Median)		43 weeks	4.5 weeks	81 weeks *
Time Since Initial Injury (Mode)		3 weeks	3 weeks	52 weeks
Gender (*n*)	Female	10	7	3
	Male	21	8	13
Race (*n*)	Black	9	4	5
	White, Hispanic	6	3	3
	White. Non-Hispanic	16	8	8
Level (*n*)	Paraplegia	13	7	6
	Tetraplegia	18	8	10
Mobility (*n*)	Power Wheelchair	15	7	8
	Manual Wheelchair	16	8	8

* Includes follow-up injury after previous SCI.

**Table 2:** Descriptive results on the PR-SBC.

Variable		N	Mean	SD
PR-SBC		31	6.22	1.90
Yrs since Injury		31	3.45	9.11
Setting	Inpatient	15	5.52	1.64
	Outpatient	16	6.87	1.95
Injury Level	Tetraplegia	18	5.71	1.99
	Paraplegia	13	6.92	1.58
SCI Severity	AIS A/B	16	6.43	2.11
	AIS C/D	15	5.99	1.70
Mobility	Power Chair	15	5.33	1.74
	Manual Chair	16	7.05	1.70

**Table 3:** Linear regression results for the PR-SBC.

Predictor	Reference	Estimate	SE	95% CI	*p*-Value	VIF
Outpatient	Inpatient	1.51	0.64	0.18–2.83	0.027	1.16
Yrs since Injury		−0.00	0.03	−0.07–0.07	0.973	1.07
Paraplegia	Tetraplegia	0.47	0.75	−1.07–2.01	0.533	1.54
AIS C/D	AIS A/B	0.20	0.66	−1.16–1.57	0.763	1.24
Manual chair	Power chair	1.53	0.73	0.02–3.04	0.047	1.52

**Table 4:** Inter-rater reliability evidence.

Group	N	ICC (2,1)	95% CI
Sample	31	0.96	0.88–0.98
Inpatient	15	0.92	0.70–0.97
Outpatient	16	0.98	0.94–0.99

**Table 5:** Relationship between PR-SBC and SCI-FCS.

Group	N	Pearson *r*	*p*-Value
Sample	31	−0.51	0.003
Inpatient	15	−0.41	0.13
Outpatient	16	−0.59	0.02

**Table 6:** Psychometric properties of the SCI-Falls Concern Scale.

Ref	Average SCI-FCS Score (16 to 64)	Test–Retest Reliability	Inter-Rater Reliability	Internal Consistency Reliability	Validity
[20]	Median = 24	ICC = 0.93		α = 0.92	Participants who fell less had higher scores
[21]		ICC = 0.81		α = 0.97	
[22]	Mean = 17 + 6		ICC = 0.97	α = 0.83	*r* = 0.56 with WheelCon-Mi-I
[23]	Mean = 23 + 7	PA ^1^ > 70% (all but 1 item) ICC = 0.83		α = 0.88	SDC ^2^ = 7
[24]	Median = 21			α = 0.95	Higher scores among persons with a fear of falling

^1^ Percent Agreement. ^2^ Smallest detectable change.

## Data Availability

The data presented in this study are available on request from the corresponding author. The data are not publicly available due to privacy and ethical restrictions.

## Abbreviations

The following abbreviations are used in this manuscript:

CARF	Commission on Accreditation of Rehabilitation Facilities
ICC	Intraclass Correlation Coefficient
PR-SBC	Patient Reported Sitting Balance Confidence Scale
SCI	Spinal Cord Injury
SCI-FCS	SCI Falls Concern Scale
SCIM III	Spinal Cord Independence Measure (SCIM III)

## Appendix A

**Patient Reported-Sitting Balance Confidence Interview Instrument**

**Demographic Information**

- Time Since Injury/Illness: ________ months or _____ yrs
- Age:____________
- Gender: (check one)
  □ Male
  □ Female
  □ Nonbinary
  □ Prefer not to answer
  □ Other
- Please check which types of equipment you are currently using (check all that apply):
  □ Transfer board
  □ Power wheelchair
  □ Manual wheelchair
  □ Hospital bed
  □ Mechanical lift
  □ Wheelchair accessible vehicle

Outcome Measure

For each task below, please indicate your level of confidence in doing the activity without losing your balance or becoming unsteady. Rate your confidence on a scale from 1 (Not at all) to 10 (Completely confident). Rate your confidence in your usual environment and with your usual assistive device(s). If you do not currently do the activity in question, try and imagine how confident you would be if you had to do the activity. If you normally use an assistive device to do the activity or hold onto someone, rate your confidence as if you were using these supports. If you have a difference in strength between your right and left sides, score the lower side.

1 (Not at all)  2  3  4  5  6  7  8  9  10 (Completely)

Scoring: Average score across completed item responses.

**Sample Scoring**			
**Tasks**	**Confidence Score**	**N/A if Not Applicable**	**Is Assist Needed?**
Lie down and sit up on a regular bed	7		✔
Push or drive your wheelchair on uneven surfaces (e.g., outdoors, ramps)		N/A	

Interview Instrument

	**Confidence Score**	**N/A if Not Applicable**	**Is Assist or Another Person Needed?**
Home/Clinic Tasks			
Lie down and sit up on your bed			
Complete basic activities of daily living when sitting without back support (e.g., dressing on the edge of your bed)			
Hold an object with both hands			
Lean side to side (e.g., placing a transfer board, for pressure relief, toileting)			
Transfer between even surfaces			
Transfer between unlevel surfaces of at least 2” different, up to 5” different			
Push, drive, or ride in your wheelchair on level surfaces (e.g., within your home)			
Push, drive, or ride in your wheelchair on uneven surfaces (e.g., outdoors, ramps)			
Reach forward for an object at shoulder height far enough away that you would leave the back support of a chair			
Pick up an object from the floor and return to upright sitting			
Complete household tasks (e.g., meal preparation, laundry, housework)			
Get up from the floor			
Transfer in a wet environment, such as after bathing or when outdoors in the rain			
Community-based Tasks			
Complete recommended exercise for health (strength, cardiovascular and/or flexibility)			
Participate in desired work, volunteer or school activities			
Engage in preferred hobbies or leisure activities			
Travel in a vehicle			
Interact with your friends/child(ren)/pet			
Stay overnight somewhere other than your home (i.e. hotel, family’s home)			
Total Score			
Total Items Scored			
Average Confidence Total			

## Appendix B

Linear regression results for the PR-SBC excluding the 50-year post-injury observation.

**Predictor**	**Reference**	**Estimate**	**SE**	**95% CI**	***p*****-Value**
Outpatient	Inpatient	1.00	0.75	−0.56 to 2.55	0.199
Yrs since Injury		0.14	0.12	−0.11 to 0.39	0.245
Paraplegia	Tetraplegia	0.63	0.75	−0.91 to 2.18	0.406
AIS C/D	AIS A/B	−0.07	0.69	−1.49 to 1.35	0.920
Manual chair	Power chair	1.58	0.73	0.08 to 3.08	0.039
